# Role of WDR5 in breast cancer prognosis

**DOI:** 10.17179/excli2019-2062

**Published:** 2019-12-16

**Authors:** Regina Stoeber

**Affiliations:** 1 Leibniz Research Centre for Working Environment and Human Factors (IfADo)

## ⁯⁯⁯

***Dear Editor,***

Recently, Punzi and colleagues published a study about the role of WDR5 in breast cancer metastasis (Punzi et al., 2019[17]). WDR5 is involved in epigenetic regulation complexes and has been reported to influence the expression of numerous genes, including N-cadherin, Snail1 and vimentin (Aho et al., 2019[1]; Ford and Dingwall, 2015[6]; Wu et al., 2011[25]; Chen et al., 2017[5]; Tan et al., 2017[23]). Several studies suggested WDR5 as a therapeutic target (Ye et al., 2019[26][27]; Macdonald et al., 2019[14]; Aho et al., 2019[1]; Zhang et al., 2018[28]; Lu et al., 2018[13]). In their present work, Punzi and colleagues report that downregulation of WDR5 by shRNA in breast cancer cells antagonizes the epithelial-to-mesenchymal transition through re-differentiation and reduces metastasis in a mouse model (Punzi et al., 2019[17]). Moreover, an association of high WDR5 expression with shorter metastasis-free survival was observed in a cohort of breast cancer patients (Punzi et al., 2019[17]). A further important finding of this study is that WDR5 activates TGFβ in breast cancer and that targeting the WDR5-TGFβ axis by a small molecular inhibitor reduces the migratory potential of breast cancer cells. Metastasis of breast cancer is a complex process (Loi et al., 2019[12]; von Minckwitz et al., 2019[24]; Gogiashvili, 2018[8]; Stoeber, 2017[22]). Besides epithelial-to-mesenchymal transition, genes involved in proliferation (Schmidt et al., 2018[19]), immune cell infiltration (Schmidt et al., 2012[18], 2018[19]; Heimes et al., 2017[9][10]; Godoy et al., 2014[7]), oxidative stress response (Cadenas et al., 2010[2], 2014[3], 2019[4]; Hellwig et al., 2016[11]) and inflammatory factors (Mattsson et al., 2015[16]; Sicking et al., 2014[20]) are of relevance; also key enzymes of phosphocholine metabolism have been shown to control breast and ovarian cancer metastasis (Marchan et al., 2017[15]; Stewart et al., 2012[21]). It will be interesting to learn in the next years, whether WDR5 targeting compounds can be identified that have a perspective to be tested in clinical studies. 
